# *Ex vivo* biomechanical investigations of combined extra- and intracapsular stabilization in canines with cranial cruciate ligament deficiency

**DOI:** 10.3389/fvets.2024.1336797

**Published:** 2024-06-12

**Authors:** Cheng-Yuan Sun, Cheng-Chung Lin, Ching-Ho Wu

**Affiliations:** ^1^Institute of Veterinary Clinical Science, School of Veterinary Medicine, National Taiwan University, Taipei, Taiwan; ^2^Department of Electrical Engineering, Fu Jen Catholic University, New Taipei City, Taiwan

**Keywords:** cranial cruciate ligament deficiency, intracapsular reconstruction, combined extra-and intracapsular reconstruction, three-dimensional kinematics, gait

## Abstract

Intracapsular reconstruction (ICR) has long been recommended as a treatment for cranial cruciate ligament deficiency (CCLD) in dogs, but it has fallen out of favor due to its inferior long-term functional outcomes. These outcomes may be attributed to the poor stiffness and strength of the graft in the early period before ligamentization is completed. Additional placement of extracapsular sutures to mechanically protect the graft during the ligamentization process may be a viable method to address this problem. However, the biomechanical effect of this combined surgical approach remains unknown. This study aimed to evaluate the 3D kinematics of the CCLD stifle in dogs in response to ICR and combined extra- and intracapsular reconstruction (CEICR). Twelve hindlimbs were collected from nine cadavers of mature dogs. The limbs were tested using a custom-made testing apparatus that reproduces their sagittal plane kinematics during the stance phase. Four statuses of stifle joints were tested, namely, (a) cranial cruciate ligament (CCL) intact; (b) CCLD; (c) CCLD stifle stabilized by CEICR; and (d) CCLD stifle stabilized by ICR only. Three-dimensional stifle kinematics at the 5 instances of the stance phase were measured with an optoelectronic system. The results showed that ICR marginally corrects the increased adduction, internal rotation, and caudodistal stifle joint center displacement that occur as a result of CCLD. CEICR led to better restoration of the stifle kinematics, especially with respect to the internal rotation and cranial translation stabilities. Furthermore, CEICR only resulted in minor excessive restraints on other motion components. The findings indicated that the additional lateral fabellotibial suture offers immediate stability to the stifle, consequently lowering the risk of graft over-elongation in the short term postoperatively. Considering the propensity for the extracapsular suture to degrade over time, further *in vivo* studies are warranted to explore the long-term effects of the CEICR procedure.

## 1 Introduction

Cranial cruciate ligament deficiency (CCLD) is the most common cause of lameness in dogs (1–3). Various risk factors may be associated with the pathogenesis of CCLD, including epidemiological factors (e.g., age, body weight, genetic predisposition, gender, and neuter status) (4), stifle conformational factors (e.g., excessive tibial plateau angle and intercondylar stenosis) (5, 6), and factors related to the composition of the CCL itself (e.g., blood supply and extracellular matrix composition) (7). Numerous treatments have been introduced, most of which restore stifle stability rather than repair the deficient CCL. While intracapsular reconstruction (ICR) has been the most widely used surgical intervention for anterior cruciate ligament rupture in the human knee joint for decades (8), current approaches for stabilizing the canine stifle joint largely rely on osteotomy or extracapsular stabilization (ECS) procedures (9). However, recent studies have shown that ECS may not effectively prevent the progression of osteoarthritis postoperatively (10–13) or fully restore normal limb functions (14, 15). This may be attributed to the inability of ECS to fully restore the biomechanical characteristics of the native stifle in the absence of an intact CCL.

Some studies have indicated that ECS may result in suboptimal stifle kinematics, as anchor points outside the joint are not close to isometric (16, 17), which can lead to excessive lateral translation and external rotation of the tibia with respect to the femur (18). Conversely, tibial osteotomy sometimes exhibits a restricted capacity to impede excessive internal rotation (19), and its ability to consistently eliminate femorotibial translation also varies (15). Although ICR aims to restore the anatomy and function of the CCL, inferior long-term functional outcomes have been observed when compared to other stabilization procedures for the treatment of CCLD (20). Insufficient strength of intracapsular grafts has been reported in the early period of treatment (21), which can further lead to stifle instability and progressive cartilage degeneration (22). While the success of ICR in the human knee (23) highlights the potential benefits of this procedure in restoring normal stifle biomechanics in canine CCLD, further investigation is necessary to optimize the ICR procedure and improve the durability of grafts used in the treatment of CCLD among canines. Therefore, the continued exploration of ICR as a valid treatment for CCLD among canines is warranted.

To address the poor strength of fascia lata grafts, some studies have proposed adding an extracapsular suture to mechanically protect the graft during the healing process (24). The addition of an extracapsular suture combined with the ICR has been suggested to increase the stability of the stifle joint, thereby reducing the risk of graft overloading in the early stages of treatment. Moreover, since the ECS procedures may lead to increased contact pressure in the lateral joint compartment (25) and excessive external rotation and abduction of the stifle joint (17) due to the alignment and over-tension of the extracapsular suture, combining the extracapsular suture with ICR may decrease the tension needed for adequate stifle stability, potentially alleviating the negative effects on the biomechanical environment of the joint caused by the artificial sutures.

Outcome evaluations of surgical treatments for CCLD have been conducted by retrospective studies (26), *in vivo* limb function evaluation with force plates (20), arthroscopy (24, 27, 28), osteoarthritis scoring (27, 28), computer model prediction (29), and *ex vivo* biomechanical studies (30, 31). Among these methods, *ex vivo* biomechanical testing allows for direct experimental exploration of the effects of diseases or therapeutic factors on stifle kinematic responses. In recent years, various *ex vivo* models have been built to simulate weight-bearing gait scenarios and to investigate femorotibial translation under specific loadings or at specified joint angles or to measure the internal contact forces on the meniscus (25, 32, 33). To our knowledge, only two studies have investigated the effects of extracapsular suture on canine stifle biomechanics using a weight-bearing model reproducing the stifle kinematics during the simulated stance phase (SP) in a walking gait cycle (31, 34). The biomechanical effects of ICR combined with an additional extracapsular suture on stifle stability remain unclear.

The objectives of the present study were to develop a testing apparatus that enables the simulation of quasistatic stifle kinematics of dogs during the SP in a walking gait and to evaluate the 6 degrees-of-freedom kinematics of the stifle joint response to ICR and combined extra and intracapsular reconstruction (CEICR) among dogs with CCLD using the testing apparatus.

## 2 Materials and methods

### 2.1 Specimen preparation

All procedures in the study were approved by the Institutional Animal Care and Use Committee of National Taiwan University (IACUC number: B202000070). Fifteen pelvic limbs (*n* = 15) were collected from 9 skeletally mature medium-breed dogs weighing 15 to 25 kg (body condition score within 4–6/9). All dogs died of reasons unrelated to the study. Each cadaver underwent physical and orthopedic examinations to rule out any gross deformity or orthopedic disease. Orthogonal radiographs of the limbs were taken to exclude specimens with stifle pathology or excessive tibial plateau angle (TPA > 35°). A TPA exceeding 35° is considered indicative of tibial deformity and may render the specimens unsuitable candidates for the proposed procedure. Selected limbs were disarticulated from the coxofemoral joint and were wrapped in a saline (0.9% NaCl) solution-moistened towel, sealed in a plastic bag, and stored at −20°C until use.

**Figure 1:**
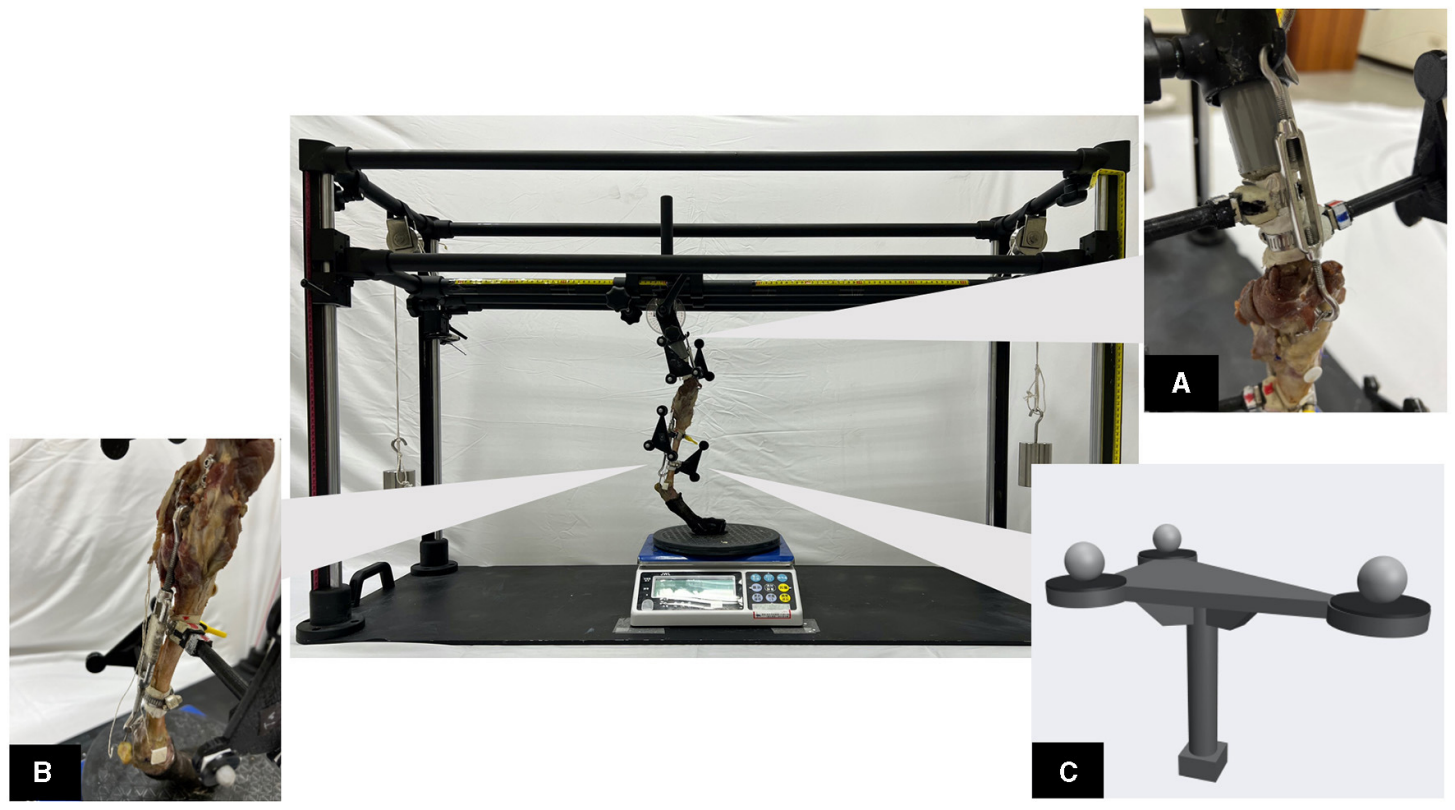
The custom-made testing apparatus with a loaded specimen in place. **(A)** The proximal part of the specimen in oblique view. The proximal end of the femur was sectioned and cemented into PVC pipes. A turnbuckle connects the artificial hip joint and patella, which mimics the quadriceps muscle mechanism. **(B)** The second turnbuckle connects the caudo-distal femur and calcaneus, which mimics the gastrocnemius muscle mechanism. **(C)** A 3D-printed tracking marker plate, with the reflective marker in place, was used for skeletal kinematics tracking. The tracking marker plate was equipped with a neodymium magnet, enabling it to be freely assembled or dissembled from the hose clamp. The hose clamp, in turn, was securely attached to the specimen during the data collection process.

Before specimen preparation, the specimens were thawed at 4°C for 24 h. The skin of the femur and tibia was removed. Soft tissues were kept moist by being sprayed with a saline (0.9% NaCl) solution repeatedly throughout the specimen preparation and experiment. A strip of fascia lata graft was harvested after skin removal, with its distal attachment of the graft left intact. The graft was then wrapped in gauze soaked with saline (0.9% NaCl) solution to protect the graft and to keep the graft moist. Next, all the muscles of the specimen were removed, with the soft tissue around the stifle joint (patellar ligament, CCL, caudal cruciate ligament, collateral ligaments, stifle joint capsule, menisci, and the muscles surrounding the stifle joint) as well as the soft tissues including the skin distal to the talocrural joint carefully preserved. The proximal end of the femur was sectioned and cemented into PVC pipes (Figure 1A).

In each specimen, two turnbuckles were used to mimic the muscle mechanism. The first turnbuckle was used to mimic quadriceps muscle function. A 1.0-mm K-wire double loop was passed through the widest portion of the patella in a lateromedial direction and connected to a turnbuckle. The other end of the turnbuckle was attached to a hook on the artificial hip joint during the experiment (Figure 1A). The second turnbuckle mimics the gastrocnemius muscle function, which connects to a short string-of-pearl plate (SOP plate) placed in the caudal aspect of the femoral metaphyseal region. A 1.0-mm K-wire double loop passed transversely through the calcaneal tuber (Figure 1B).

Reflective markers were fixed to the femur and tibia for kinematic data acquisition. The markers were divided into 2 categories: anatomical markers (*n* = 8) and tracking markers (*n* = 12). The anatomical markers were affixed to the bony landmarks, namely, the greater trochanter, lateral and medial femoral condyle, proximal and distal tibial crest, lateral and medial malleolus, and fibular head. These markers were used to determine the local coordinate system (LCS) of the femur and tibia. The twelve tracking markers were attached to four 3D-printed “tracking marker plates” (Figure 1C). The tracking markers were used to track the 3D skeletal poses during the tests. Since the marker plates influence the conduct of surgery, they were made to be capable of being attached to and detached from a hose clamp fixed to the specimen via a detachable mechanism.

### 2.2 Testing apparatus

A custom-made testing apparatus (Figure 1) was designed to simulate the SP of a walking gait for canine hindlimbs. The PVC tube embedded with the specimen was mounted onto an artificial hip joint on the upper platform. The angle of the artificial hip joint can be adjusted in the sagittal plane, which corresponds to different hip flexion angles at different instances of the SP. Four vertical aluminum rods and four linear slide rails enable the upper platform to displace in the vertical direction. A rail system on the upper platform also allows its displacement along the cranial-caudal and lateral-medial directions. To simulate different weight-bearing scenarios, the vertical weight can be adjusted by placing different weights above the platform and by hanging weights with a pulley system on both sides of the testing apparatus. At the bottom plate, a rotary disc allowing unconstrained internal-external rotation of the distal limb was placed between the footpad and an electronic scale (Jadever^®^ Scale Co., Ltd., Xiamen, China), which recorded the vertical forces applied to the specimen.

### 2.3 Experimental protocol

The testing apparatus was placed at the centroid of the experiment space in which an optical motion capture system (MoCap) was equipped with 9 infrared cameras (6 Bonita 10 cameras and 3 Vero v2.2 cameras, VICON, Oxford Metrics, UK) placed surrounding the testing apparatus. For each specimen, to obtain the fixed spatial transformations between the anatomical and tracking markers, a subject calibration procedure was executed by recording two sets of markers simultaneously. The transformations enable us to derive the anatomical marker coordinates based on the tracking markers. After the completion of subject calibration, the anatomical markers were removed as they impeded the subsequent surgical procedure.

Based on a preliminary study on gait data measurement, the displacement of the hip joint center relative to the paw position and the flexion angle of the hip joint were quantified. The vertical component of the ground reaction forces normalized to the body weight (% BW) was obtained from the study by DeCamp (35). The data selected at each 25% interval of SP were taken as the reference for the placement of the specimens, with a total of five instants. The cranial-caudal position and the vertical height of the upper platform, the weights placed on the testing apparatus, and the tightness of turnbuckles were adjusted until the slack was eliminated with the hindlimb standing stably and the hip joint position and angle and the electronic scale reading underneath were consistent with the reference data.

Each specimen was tested under four different statuses, namely, CCL intact, CCLD, CCLD stifles repaired by CEICR, and CCLD stifles repaired by ICR only. After the completion of biomechanical testing on CCL intact status, the transection of the CCL was performed through a minimal caudal arthrotomy. The entire CCL was completely transected from its origin, which was confirmed by a cranial drawer test. The remnants of the CCL at its tibial insertion point were also transected through a para-patellar lateral arthrotomy. The joint capsule was then closed with a simple continuous pattern. Biomechanical testing was thereafter performed again under CCLD status.

For the CEICR procedure, a 3.5-mm or a 4.5-mm bone tunnel was created, starting from the center of the CCL femoral footprint to the lateral cortex of the distal femur diaphysis. The proximo-distal level of the bone tunnel exit was at the fabella. The bone tunnel size was chosen to maximize the tunnel diameter while ensuring it remained within 40% of the bone diameter. Alligator forceps were passed through the joint space in a caudo-cranial direction and passed underneath the cranial intermeniscal ligament. The graft was passed under the cranial intermeniscal ligament and then pulled through the joint. Next, the alligator forceps were passed through the femoral bone tunnel in a retrograde fashion, with which the graft was pulled through the bone tunnel. Preconditioning of the graft was performed by applying a 4-kgw weight to the end of the suture for 10 min, which eliminated the laxity of the graft. While tension was maintained on the graft and the specimen showed no cranial drawer sign, the graft was entrapped and secured with a spiked washer (Jing Jiuh Technology Co., Ltd., Taiwan), which was placed with a 3.5-mm cortical screw. The incised joint capsule was approximated with a 2-0 polydioxanone suture with a simple continuous pattern. Afterward, the lateral fabellar suture (LFS) technique was performed using an 80-lb test nylon leader line (NLL, Securos Inc., United States) secured with a titanium nitride-coated stainless steel crimp clamp (80# TiN Coated Crimp Clamp, Securos Inc., United States). The absence of cranial drawer motion was verified manually after the surgery. After the completion of testing under the CEICR status, the nylon leader line was cut and removed, followed by biomechanical testing under the ICR status. Each test session required approximately 30 to 40 min to complete. To mitigate soft tissue desiccation, intermittent saline misting was applied between each session. A post-test examination was also conducted to ensure that there was no occurrence of graft failure during the experiment.

### 2.4 Kinematic analysis

The marker data collected from MoCap were manually labeled with Nexus software and transferred to a self-developed motion analysis program based on MATLAB (R2020a, Mathwork, Inc., United States). The LCS of the femur and tibia were first determined using anatomical markers following the conventions published in Fu et al. (36). The kinematics of the stifle joint with six degrees of freedom were analyzed at five specific instants of SP. The joint rotations were expressed in terms of flexion/extension, abduction/adduction, and internal/external rotation angles. Furthermore, the positions of the stifle joint center (SJC), precisely defined as the midpoint between the epicondylar markers, were described in tibial LCS. The kinematic data were averaged across three trials for each time instant and condition. The translation of the SJC was defined as the linear displacement in its position relative to that in the state of CCL intact and at 50% SP.

### 2.5 Statistical analysis

The Shapiro–Wilk test was used to assess the normality of each parameter's distribution. Due to the non-normality of the data, Friedman's test was used to rank the grouped data and identify significant differences in flexion/extension, abduction/adduction, and internal/external rotation angles and cranial/caudal, proximal/distal, and lateral/medial translations among different joint statuses. The Wilcoxon signed-rank test was conducted for *post hoc* analysis, with a significance level of α = 0.05.

## 3 Results

### 3.1 Subjects

Fifteen hindlimbs were collected from ten adult mongrel breed dogs. Seven right and five left hindlimbs were included in the final analysis, as three limbs were excluded due to technical problems (e.g., marker displacement during the experiment and failure of the implants mimicking the muscle). The body weight ranged from 15.0–19.5 kg (mean ± SD: 17.52 ± 1.64 kg).

### 3.2 Kinematic data

**Figure 2:**
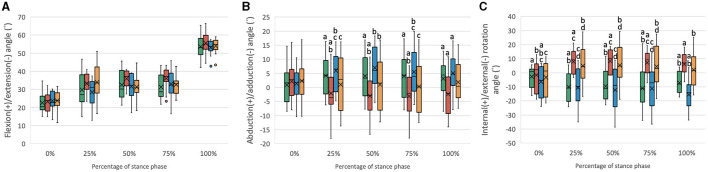
Boxplots of the distribution of the **(A)** flexion/extension angles, **(B)** abduction/adduction angles, and **(C)** internal/external rotation angles for stifle joints with intact CCL (Intact), transected CCL (CCLD), and repaired with combined extra and intracapsular reconstruction (CEICR) and intracapsular reconstruction (ICR). The median is represented by the central lines, and the mean values are represented by the cross; the edges of the boxes represent the upper and lower quartiles of the distributions; the solid dots represent outliers; and the upper and lower extreme ranges are indicated by the whiskers. Within the same percentage, groups with the same letters (a-d) differ significantly (*p* < 0.05).

**Figure 3:**
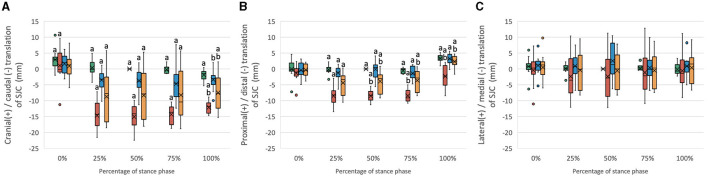
Boxplots of the distribution of **(A)** cranial/caudal translation, **(B)** proximal/distal translation, and **(C)** medial/lateral translation of the SJC for stifle joints with intact CCL (Intact) and transected CCL (CCLD) and repaired with combined extra and intracapsular reconstruction (CEICR) and intracapsular reconstruction (ICR). The median is represented by the central lines, and the mean values are represented by the cross; the edges of the boxes represent the upper and lower quartiles of the distributions; the solid dots represent outliers; and the upper and lower extreme ranges are indicated by the whiskers. Within the same percentage, groups with the same letters (a-d) differ significantly (*p* < 0.05).

**Figure 4:**
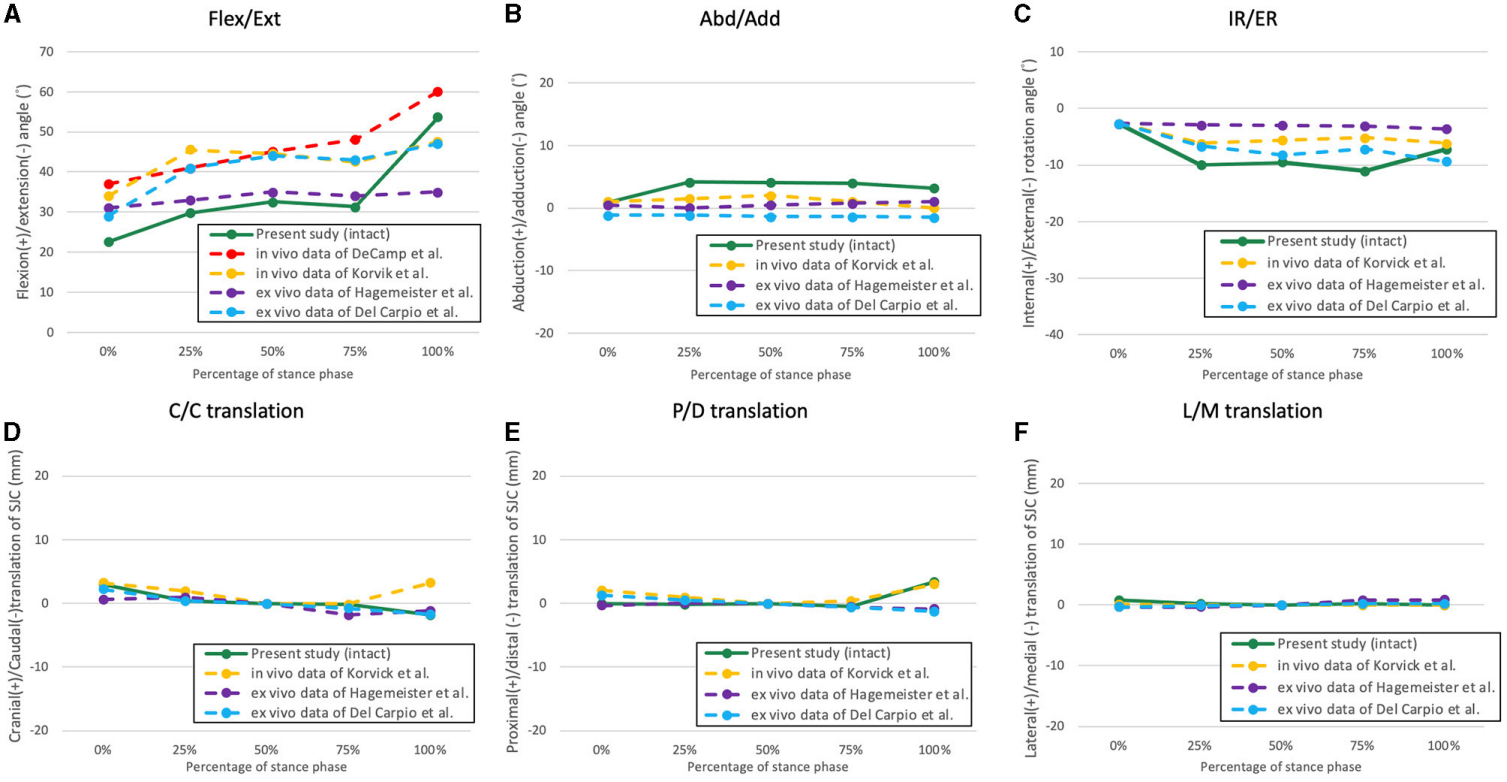
The averaged kinematic curves of the intact stifle throughout the simulated stance phase obtained from the present study and former studies. **(A)** Flexion/extension; **(B)** abduction/adduction; **(C)** internal/external rotation; **(D)** cranio-caudal translation of the SJC; **(E)** proximo-distal translation; **(F)** latero-medial translation.

The six-degree-of-freedom kinematic data of the stifle with intact CCL and CCLD and repaired with CEICR and ICR are summarized in Figures 2, 3. The boxplots indicated the distributions and patterns of the tibial rotations with respect to the femur and SJC translations relative to the tibia during the progression of the SP for all the specimens. The kinematic data of the intact stifles in our study align closely with findings from previous *in vivo* studies (35, 37). Throughout the SP, we observed a progressive rise in the flexion angle and minimal caudodistal translation of the SJC. On the other hand, both the abduction angle and internal rotation angle peaked during the mid-stance phase (Figure 4).

#### 3.2.1 Rotation component

The SP begins with a mean stifle flexion angle of approximately 20°-25°. As the SP progressed, the flexion angle exhibited a gradual increase, eventually reaching an approximate range of 53°-56° (Figure 2A). No statistically significant differences were observed among the various stifle statuses. The primary objective of this study was to evaluate the coupled motions in the other five kinematic components at the same flexion angle. Given the consistent position of the hip joint center and hip joint angle, it was reasonable that there would be no significant differences in stifle flexion/extension angles among different stifle statuses.

The mean abduction/adduction angle of the intact stifle remained relatively constant at approximately 0°-5° throughout the SP. However, following the transection of the CCL, a significant increase in adduction (mean value: −5°-0°) was observed during the 25%−100% SP. Conversely, after CEICR, the stifle adduction was restored during the 25%−75% SP, with no statistically significant differences when compared to those observed in the stifle joints with an intact CCL. However, toward the end of the SP, a significant increase in abduction of approximately 2° was observed compared to the CCL intact stifle. The overadduction of the CCLD stifle was also corrected by ICR without causing excessive abduction throughout the SP (Figure 2B).

Throughout the SP, the CCL intact stifle exhibited a slight external rotation, with an average magnitude of 7° at the end of the SP. The transection of the CCL resulted in a significant increase in IR, with the mean IR angles ranging between approximately 5° and 10° during the 25%−100% of the SP. The CEICR restored the excessive IR during the weight-bearing period of the SP. However, at the beginning and end of the SP, when no weight was applied to the limb, the CEICR led to a mean external rotation angle of approximately 14°, which was significantly greater than that observed in the intact stifle. The ICR failed to eliminate the excessive IR caused by CCLD.

#### 3.2.2 Translation components

The SJC of the CCL intact stifle displayed a minor cranial-to-caudal shift during the SP. The transection of the CCL resulted in significant 10- to 15-mm increases in caudal translation of the SJC throughout the 25%−100% SP (Figure 3A). Both the CEICR and ICR were observed to ameliorate the excessive caudal translations of the SJC, as evidenced by the mean caudal translations of up to −4.6 mm and −8.2 mm, respectively (Figure 3A). During 0% and 100% SP, no significant difference was found between CEICR and CCL intact stifles.

The stifle with intact CCL exhibited subtle proximal–distal motion throughout the entire SP. However, following the transection of the CCL, significant distal translations ranging from 1.2 mm to 8.4 mm were observed in 25%−100% SP (Figure 3B). After CEICR, while significant distal translation at 25% SP was still detected, the translation differences with respect to those in CCL intact stifles were <1.5 mm. On the other hand, the ICR enabled a reduction in the distal translation caused by the CCL transection, but it was not capable of fully eliminating the excessive distal translation throughout the 25%−100% SP. The means of proximal/distal translational differences exceeded 2 mm throughout this period (Figure 3B).

Minor lateral translation in the CEICR group and medial translation in the CCLD group were observed, but no significant difference was found among groups throughout the SP.

## 4 Discussion

Some *in vivo* studies have documented the kinematics of the entire stance phase of canines (35, 37–39); however, to our knowledge, only two studies have reproduced the entire canine stance phase with a testing apparatus (31, 34). In the present study, we aimed to reproduce the sagittal plane kinematics of the canine hindlimb utilizing data collected from a preliminary study. The expectation was to reproduce stifle flexion angles that closely resembled those of a healthy subject under near physiological conditions using a quasidynamic model. In addition, we compared the reproduced stifle flexion angles with those from *in vivo* and *in vitro* studies (Figure 4) and were found to be similar in magnitude and trend, albeit with minor discrepancies (35, 37). The discrepancies observed may have originated from variations in body conformation and motion tasks that were measured across the canine subjects. For example, Korvick et al. recorded stifle kinematics during the trotting gait (37), while our study aimed to replicate the walking gait. Thus, in accordance with the similar trends and angle magnitudes observed, we consider that our *ex vivo* model can reproduce hindlimb motions closely resembling the physiological kinematics during the SP of a walking gait.

This is the first study to document the 3D kinematics of the stifle treated with ICR and CEICR procedures during the weight-bearing SP of a gait. Compared to the intact stifle, transection of the CCL led to a significant increase in adduction, internal rotation angle and caudo-distal displacement of the SJC throughout the 25%−100% SP. In general, kinematic deviations caused by CCLD were majorly corrected by the CEICR procedure. Despite significant differences in some motion components detected, the magnitude of kinematics deviations with respect to the CCL intact stifle were diminished. Conversely, the ICR was only able to marginally correct the kinematic deviations as a result of the CCLD.

In comparison to the CCL-intact stifle, a significantly increased adduction was observed during the 25%−100% SP after CCL transection. Notably, while the medial collateral ligament and the lateral collateral ligament are the major stabilizers of stifle abduction/adduction (40), the role of the CCL in stifle abduction/adduction has rarely been studied. In a recent *ex vivo* SP simulation model conducted by Del Carpio et al. (31), increased adduction was also observed following CCL transection, with reported values similar to those of our study. To our knowledge, this finding has not been demonstrated in any *in vivo* studies. Since dogs tend to modify their gait to adapt to CCLD (37), differences in weight-bearing and stifle flexion angle between *in vivo* and *ex vivo* conditions could provide a plausible explanation for the disparities observed. A prior report indicated that an increase in the flexion angle is associated with an increase in abduction in the stifle joint (38). Therefore, considering that the stifle is typically more flexed during the SP of *in vivo* conditions (37), less adduction was expected.

Although adduction was not entirely eliminated by the ICR treatment, no significant difference was observed between the CCL intact and the ICR status. Compared with the impact on internal/external rotation, proximal/distal translations, and cranial/caudal translations, the ICR exhibited a satisfactory restorative effect on abduction/adduction. In contrast, the CEICR procedure resulted in mild abduction, with significant differences observed during the non-weight-bearing SP (i.e., 0% and 100%). This finding was consistent with most of what was found in the previous studies (17, 18), which reported that LFS can cause significant abduction of the stifle. However, the increased magnitude of abduction in the CEICR procedure in the present study (i.e., 2°) was much less than that reported by Aulakh et al. (i.e., 6°) (18). It is likely that less tension was applied in the LFS fixation during the CEICR procedure, as the force required to counteract the cranial tibial thrust is shared by both the ICR and LFS suture, leading to less abduction than in previous LFS procedures. Further studies are warranted to better understand the effect of suture tensioning on 3D kinematics in LFS techniques and the effect of combining surgery on the tension of the LFS suture.

Compared to the intact stifle, CCL transection resulted in notable internal rotation during 25%−100% of the SP. Previous studies have well-established the role of the CCL in internal/external rotational stability (31, 37, 41). In particular, the CCL and the caudal cruciate ligament intertwine to prevent excessive tibial internal rotation (42). Therefore, a CCLD stifle commonly exhibits excessive internal rotation. The ICR procedure failed to correct the excessive internal rotation caused by CCLD (Figure 3). The insufficient torque provided by the graft near the rotation center of the stifle joint is thought to be the major issue related to the persistent internal rotation instability with the ICR. This may be due to insufficient graft stiffness. In contrast, with the CEICR procedure, the internal rotation caused by CCLD was corrected. The minor external rotation noticed in the 0% and 100% SP with CEICR may also be attributed to the additional LFS suture. Previous studies have suggested that external fixation may cause excessive external rotation (17, 22), but the external rotation caused by CEICR was less prominent, possibly due to differences in suture tensioning.

The present study found that transection of the CCL resulted in significant caudal and distal translation of the SJC in the 25%−100% SP, which was expected due to the role of the CCL in resisting cranial tibial thrust. We also found that the cranial and distal translation values were slightly higher than those in previous studies (31, 37), which could be due to higher peak vertical forces applied and different measurements used. Notably, the evaluation of SP kinematics is considered more influential for understanding the pathological mechanism of CCLD, as the dynamic components (e.g., muscle forces) can compensate for excessive cranial joint laxity during the swing phase, and kinematic abnormalities caused by CCLD were more prominent during the SP (37). In contrast to the *ex vivo* model, less cranial translation was observed in *in vivo* experimental scenarios (37) because excessive cranial translation can lead to discomfort and will be intentionally avoided by applying less weight on the affected limb.

The failure of the ICR procedure to fully restore tibial cranial instability and excessive internal rotation is an important finding of the present study. The restoration of cranial/caudal stability is indeed a crucial objective of CCLD treatments, and the inability of the ICR procedure to achieve this goal highlights the challenges associated with treating this condition. This consequence may be associated with poor graft stiffness or fixation strength. With CEICR stabilization, stifle kinematics were better restored at the 5 instants of SP. However, mild caudal translation of the SJC was still observed at 25%−75% SP, but the increase in caudal translation was <4.5 mm throughout the SP. Future studies could explore alternative graft selections or varied fixation methods to optimize the performance of the ICR procedure. Additionally, the investigation of incorporating ICR techniques with tibia osteotomies may be undertaken to establish a more favorable biomechanical environment for the graft.

It should be noted that the primary objective of the present study does not involve the exploration and correction of potential risk factors of CCLD, such as excessive tibia plateau angle. Given the advancements in various tibial osteotomy treatments, such as TPLO, which aim to modify the geometry of the tibial plateau and provide dynamic stabilization under weight-bearing conditions, the proposed testing apparatus appears well-suited for further investigating postoperative 3D kinematic responses to these osteotomy procedures.

Some limitations in the present study should be noted. First, dogs weighing more than 25 kg and those with a TPA exceeding 35° were excluded from the study cohort. It remains unclear whether these dogs would exhibit similar performance with the CEIAR procedure. Second, the testing sequence may influence the experimental outcomes. Our preliminary studies indicated that the ICR alone is susceptible to losing stiffness under high-loading conditions, even without clear signs of gross graft failure. To mitigate the risk of damage to the ICR, which could lead to subsequent experiments being unfeasible, we chose to conduct the CEICR before the ICR test. However, potential interferences associated with the fixed testing sequence, such as fatigue and desiccation of periarticular soft tissues, were inevitable. Our means of addressing these effects was to implement intermittent saline misting between each test session to alleviate tissue desiccation. Third, the discrepancies between the forces generated from the simplified arrangement of turnbuckles in the proposed *ex vivo* model and those produced by actual muscles remain unclear, given the absence of available data for *in vivo* individual muscle/tendon forces during gait. Furthermore, the forces required to maintain quasi-static hindlimb postures were also unlikely to agree with those required to generate dynamic motions. Given these inherent limitations associated with the *ex vivo* study design, it is imperative to further validate and assess any outcomes and potential complications related to the CEIAR procedure *in vivo*.

In conclusion, the CEIAR technique proposed in this study effectively addresses the limitations of the ICR construct alone, particularly in improving internal/external and cranial/caudal translation stabilities, while imposing minimal constraints on other motion components. This finding suggested that the additional LFS provided immediate postoperative stability to reduce the over-elongation of the graft, potentially diminishing the risk of early graft failure. As the ECS component of the CEICR is prone to degrade over time, future studies are warranted to refine the ICR procedure and investigate the long-term outcomes of the CEICR procedure.

## Data availability statement

The raw data supporting the conclusions of this article will be made available by the authors, without undue reservation.

## Ethics statement

The animal study was approved by the Institutional Animal Care & Use Committee. The study was conducted in accordance with the local legislation and institutional requirements.

## Author contributions

C-YS: Writing – original draft. C-CL: Writing – original draft. C-HW: Writing – review & editing.
